# Effects of Pallidal Deep Brain Stimulation on Speech and Swallowing in Pediatric Patients with Dystonia

**DOI:** 10.1002/mdc3.70454

**Published:** 2025-11-27

**Authors:** Katerina Bernardi, Andrea A. Kühn, Ana Luísa de Almeida Marcelino, Matthias Eckenweiler, Cornelia Rensing‐Zimmermann, Joachim K. Krauss, Joachim Runge, Rene Marquez Franco, Delia Lorenz, Monika Müller, Alfons Schnitzler, Andrea Bevot, Lidwin von Spee, Veerle Visser‐Vandewalle, Lars Timmermann, Petra Schiller, Anne Koy

**Affiliations:** ^1^ Department of Pediatrics, Faculty of Medicine and University Hospital Cologne University of Cologne Cologne Germany; ^2^ Department Human Neuroscience Sapienza University Rome Italy; ^3^ Department of Neurology and Experimental Neurology, Charité—Universitätsmedizin Berlin Freie Universität Berlin and Humboldt‐Universität zu Berlin Berlin Germany; ^4^ Department of Neuropediatrics and Muscle Disorders, University Medical Center, Faculty of Medicine University of Freiburg Freiburg Germany; ^5^ Department of Neurosurgery Hannover Medical School Hannover Germany; ^6^ Department of Stereotactic and Functional Neurosurgery, Faculty of Medicine and University Hospital Cologne University of Cologne Cologne Germany; ^7^ Department of Pediatrics University Children's Hospital Wuerzburg Germany; ^8^ Institute of Clinical Neuroscience and Medical Psychology, Medical Faculty Heinrich Heine University Düsseldorf Düsseldorf Germany; ^9^ Department of Neurology, Medical Faculty Heinrich Heine University Düsseldorf Düsseldorf Germany; ^10^ Department of Pediatric Neurology and Developmental Medicine University Children's Hospital Tübingen Tübingen Germany; ^11^ Department of Neurology University Hospital Giessen and Marburg Marburg Germany; ^12^ Institute of Medical Statistics and Computational Biology, Faculty of Medicine and University Hospital Cologne University of Cologne Cologne Germany; ^13^ Center for Rare Diseases, Faculty of Medicine and University Hospital Cologne University of Cologne Cologne Germany

**Keywords:** DBS, dystonia, Frenchay dysarthria assessment 2, speech, swallowing

## Abstract

**Background:**

Bilateral globus pallidus internus deep brain stimulation (GPi‐DBS) is a proven safe and effective treatment in certain forms of idiopathic or inherited dystonia (ID/IN). Its effects in acquired dystonia such as in dyskinetic cerebral palsy (DCP) however vary widely. The impact of GPi‐DBS on speech and swallowing, which significantly affect quality of life, remains poorly understood, especially in pediatric patients.

**Objective:**

To evaluate GPi‐DBS effects on speech and swallowing using the Frenchay Dysarthria Assessment 2 (FDA‐2), in pediatric patients with dystonia, and assess how the effects differ between DCP and ID/IN patients.

**Methods:**

This pro‐ and retrospective multicenter study analyzes speech and swallowing pre‐ and 12 months post‐GPi‐DBS using FDA‐2, including prospective data from the STIM‐CP trial and retrospective data from the GEPESTIM registry.

**Results:**

Twenty‐six patients were included (17 male, 9 female; 14 DCP, 12 ID/IN) with mean age of 12.2 years at DBS. No significant changes in FDA‐2 total scores were observed pre‐ and post‐DBS (pre: 46.3 ± 33.6; post: 46.3 ± 34.2). ID/IN patients showed consistently higher scores compared to DCP patients both pre‐ and post‐DBS (*P* < 0.005). When adjusted for age, medication, and pre‐surgical values, group differences narrowed, with minimal changes from baseline in both groups.

**Conclusion:**

GPi‐DBS did not significantly change FDA‐2 scores pre‐ and post‐DBS. Assessing speech and swallowing in pediatric patients with dystonia, impaired expressive language and/or intellectual disability is challenging. More comprehensive and patient‐centered assessment tools are needed to fully capture DBS effects on these domains in these complex disabled patients.

Dystonia is a movement disorder characterized by sustained or intermittent abnormal movements, postures, or both.^1^

Childhood‐onset dystonia is most frequently acquired, eg, due to perinatal brain damage as in cerebral palsy (CP).^2^ However, advances in next generation sequencing techniques continue to uncover new genetic causes of dystonia manifesting in early childhood.^3^ Approximately 10–15% of patients with CP have dyskinetic cerebral palsy (DCP), typically presenting with a combination of dystonia and choreoathetosis.^4^ The majority of these individuals suffer from severe physical disabilities caused by abnormal movements and postures, leading to musculoskeletal deformities. These motor impairments are frequently accompanied by other motor and non‐motor comorbidities, including cognitive impairment, speech and swallowing disturbances, as well as sleep disorders.^5^, ^6^

While acquired dystonia most commonly presents as a complex syndrome including other (neurologic and non‐neurologic) features, inherited dystonias can include isolated, but also combined (with other movement disorders) forms.^7^

The management of both, DCP and idiopathic or inherited dystonias (ID/IN), is particularly challenging, as pharmacological treatments are often ineffective or limited by medication‐related side effects^8^ (eg, sedation and respiratory depression).^9^

Deep brain stimulation of the globus pallidus internus (GPi‐DBS) is an established and safe treatment option for several movement disorders and has been proven effective in patients without a satisfactory response to pharmacological therapy, impairing generalized, inherited dystonia.^10^, ^11^, ^12^ Improvement of dystonia severity after GPi‐DBS assessed by the Burke‐Fahn‐Marsden Dystonia Rating Scale (BFMDRS), has been shown especially for children with certain forms of inherited dystonia such as DYT‐TOR1A, or DYT‐KMT2B,^13^ but also for patients with DCP.^14^ Nevertheless, in patients with DCP, effects were considerably less pronounced and showed greater variability compared to patients with ID/IN.^6^, ^15^

The BFMDRS is frequently used as the primary outcome measure in DBS studies for dystonia, however, it captures only the severity of dystonia, failing to reflect the full extent of impairment in these complex patients.^4^ It is of note, that besides hand function parents most often address improvements in communication as their main concern in the decision‐making process for DBS.^16^ Hence, speech and swallowing are perceived as very relevant for the quality of life of these patients but are only superficially assessed by the BFMDRS, highlighting the need for more comprehensive assessments. Overall, the understanding of the impact of DBS on these functional domains is still limited, especially in pediatric patients.

Although some data exist for the adult population, mainly presenting Parkinson's disease or inherited dystonia, the effects of DBS on speech and swallowing vary significantly between studies, resulting in conflicting outcomes.^17^ Some studies suggest that GPi‐DBS may have a beneficial effect on speech and swallowing function,^18^, ^19^ while others indicate that DBS may have no effect or even worsen these functions. Indeed, speech disturbances have been reported as a common side effect of the treatment, particularly as a stimulation‐induced side effect, with a prevalence of up to 12% in ID/IN.^11^, ^20^, ^21^

Moreover, there are no specific tools to assess speech and swallowing in pediatric patients with dystonia who have undergone DBS. The Frenchay Dysarthria Assessment 2 (FDA‐2) is an objective tool, commonly used in adult patients,^22^, ^23^ and has been utilized in young adults (aged 21–39 years) with DCP who have undergone DBS.^4^ This task‐based scale utilizes specific questions and targeted exercises to assess the patient's oromotor abilities and speech function. Although originally designed to study dysarthria in patients older than 12 years old, the FDA‐2 enables experienced speech therapists to extrapolate sufficient information to evaluate the patient's swallowing abilities.^24^, ^25^

This study evaluates speech and swallowing function in pediatric patients with DCP and ID/IN before and after GPi‐DBS implantation using the standardized FDA‐2 tool. We aim to determine whether GPi‐DBS affects speech and swallowing function in this patient cohort and to compare outcomes between DCP and ID/IN patients.

## Methods

### Study Design and Participants

We enrolled 26 pediatric patients from seven clinical sites, diagnosed with ID/IN or DCP, with no or unsatisfactory responsiveness to pharmacological treatment, and who had undergone bilateral GPi‐DBS for the management of dystonia.

Patients and respective pre‐ and post‐operative data were included from the prospective multicenter STIM‐CP study (NCT02097693), and from the longitudinal, retrospective GEPESTIM registry (DRKS813‐168), both approved by the ethics committee of the University of Cologne (16–03 and 13–168) and by the local ethics committees of the participating centers.

All capable patients or their legal caregivers provided written informed consent.

### Assessments

Speech and swallowing functions were assessed using standardized measures derived from the FDA‐2 scale.^26^ The FDA‐2 comprises seven sections with a total of 26 items: reflexes (three items), respiration (two items), lips (five items), palate (three items), laryngeal (four items), tongue (six items), intelligibility (three items). These sections are scored from 0 (completely abnormal) to 4 (normal) with half‐point increments for a total of nine possible scores, yielding a total score up to 104, where a higher score indicates better performance.^26^

In addition to the standard FDA‐2 analysis, we employed a categorization model based on Cardoso et al^26^ for a more comprehensive evaluation of oromotor and speech functions. This model reorganizes the FDA‐2 items into three distinct categories: (a) Oral structure at rest (respiration, lips, and tongue observation); (b) Nonverbal oromotor function, (coughing, swallowing, drooling, and various lip, palate, laryngeal, and tongue movements); and (c) Motor speech function (speech production across multiple subsystems). The possible total scores are 12, 60 and 32, respectively.

### Procedures

All patients were assessed by a speech‐language therapist and/or child neurologists who had received specific training in the FDA‐2 protocol. The assessment was performed as part of the routine baseline assessment prior to DBS surgery and then at the 12‐months post‐DBS clinical follow‐up. During this interval of time patients received continuous stimulation. Speech therapy was continued by those patients, who already had received treatment at time of recruitment, but no specific request was made to start speech therapy.

### Statistical Analysis

Continuous variables were summarized by mean and standard deviation or 95%‐confidence intervals, time variables with median and quartiles, respectively. Categorical variables were summarized by absolute and relative frequencies.

To evaluate the effect of GPi‐DBS on the outcomes measured the changes from pre‐ to post‐DBS were analyzed by paired t tests (2‐sided). Differences between the two diagnosis groups, ID/IN and DCP, were analyzed by unpaired t‐tests (2‐sided). In addition, univariate analyses of variance (ANOVA) were performed with the post‐DBS score as dependent variable and the fixed factors diagnosis group (ID/IN vs. DCP), usage of antidystonic medication (yes vs. no), age and pre‐DBS score (no interaction, type III sum of squares method; covariates appearing in the model are given below the respective table). Estimated marginal means (EMM), 95%‐ confidence intervals and *P*‐values were derived for both diagnosis groups and for the difference between groups. The modeled effects were presented together with the observed results to allow a comparison of unadjusted and adjusted results.

All analyses are essentially descriptive. Thus, no correction for multiple testing was applied. Statistical analyses were performed using the software SPSS (IBM Corp, Armonk, New York, USA). The threshold of 0.05 was set for statistical significance.

## Results

### Patient Characteristics

The study included 26 patients (17 male, 9 female), comprising 14 patients with DCP and 12 with ID/IN. The mean age at DBS was 12.2 years (range: 5–18 years). Main characteristics of the enrolled patients are presented in Table 1.

**TABLE 1:** Demographic and clinical characteristics of the included patients at DBS

	Total	DCP	ID/IN
Age			
*N*	26	14	12
Median (IQR)	12.5 (9 to 15)	12.0 (9 to 15)	13.5 (10 to 15)
Range	5 to 18	7 to 18	5 to 16
Sex, *n* (%)			
Female	9 (35)	5 (36)	4 (33)
Male	17 (65)	9 (64)	8 (67)
Dystonia, *n* (%)			
Isolated	4 (15)	0 (0)	4 (33)
Combined	22 (85)	14 (100)	8 (67)
Communication Device, *n* (%)	8 (31)	6 (43)	2 (17)
PEG, *n* (%)	2 (8)	1 (7)	1 (8)
Speech Therapy, *n* (%)	15 (577)	10 (71)	5 (42)
Anti‐dystonic Medication (post‐DBS)	13 (50)	9 (64)	4 (33)

*Note*: Results are given as median with interquartile range and range (continuous variables) or numbers and percentage (categorical variables); IQR interquartile range: first to third quartile, range: minimum to maximum.

In the ID/IN group, we identified specific genetic etiologies in 10 patients: three patients with pathogenic mutations in the TOR1A gene, the others with pathogenic variants in SGCE, GNAO1, KMT2B, ADCY5, PANK2, ANO3, and HPRT1. The remaining two patients had idiopathic dystonia with no identified cause despite commercially available genetic testing.

In the DCP group, the main etiology was hypoxic ischemic encephalopathy (HIE), either associated or not with prematurity. One patient presented with neonatal stroke and one with prematurity and neonatal sepsis. Detailed patient etiologies are reported in Table S1a.

To provide valuable context for understanding our cohort's pre‐intervention status, particularly for readers less familiar with the FDA‐2 assessment, we report descriptive data obtained through the BFMDRS speech subsection and Communication Function Classification System (CFCS)^27^ specifically for DCP patients. At baseline, the BFMDRS speech subsection revealed an average score of 3.3 for DCP patients and 1.9 for ID/IN patients (range 0–4, where 0 indicates normal speech and 4 indicates anarthria), suggesting more preserved communication abilities in the latter group. Baseline CFCS assessment for DCP patients showed an average score of 4.1 (range 1–5), similarly indicating significant communication difficulties. Our study specifically utilized the FDA‐2 as the primary outcome measure to systematically assess speech and swallowing function pre‐ and post‐DBS, as detailed in the following sections.

### Overall FDA‐2 Scores

**Figure 1:**
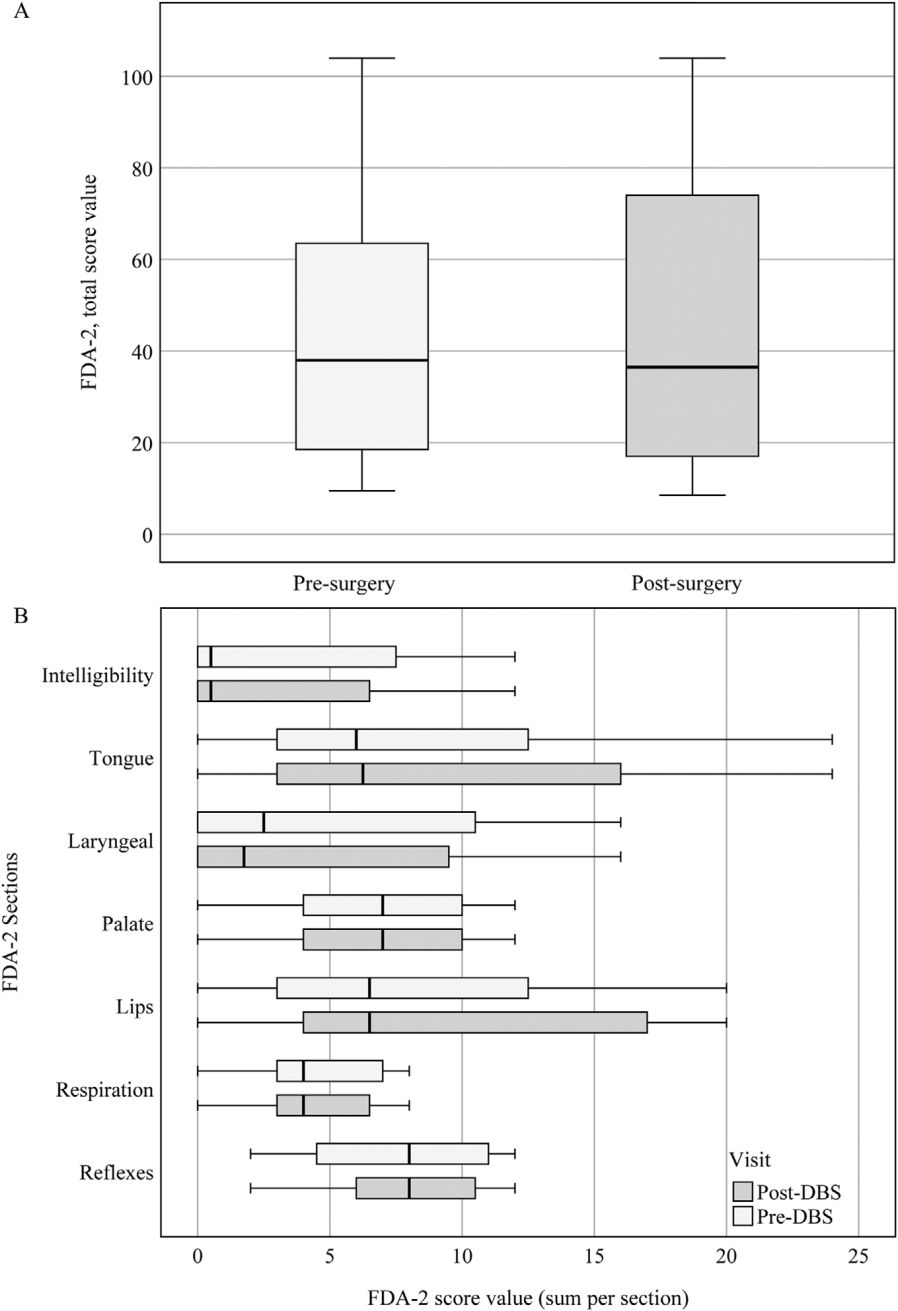
FDA‐2 total score (A) and section scores (B) of all patients taken together before surgery (pre, light gray) and 12 months after surgery (post, gray). Box plots display first and third quartile (bottom and top line of the box), median (middle line), whiskers show maximum and minimum values.

In average, for the overall cohort, there were no significant changes in FDA‐2 total scores pre‐ and post‐DBS (pre‐DBS: mean 46.3 ± 33.6, median 38, IQR 18.5–63.5; post‐DBS: mean 46.3 ± 34.2, median 36.5, IQR 17–74; change: 0.0 ± 5.2, *P* = 0.970). This was also the case for individual sections, as illustrated in Figure 1.

### Group Comparisons: ID/IN Vs. DCP

ID/IN patients consistently demonstrated higher FDA‐2 total scores compared to DCP patients, both pre‐DBS (ID/IN: 66.1 ± 35.0; DCP: 29.2 ± 21.6; *P* = 0.005) and post‐DBS (ID/IN: 67.1 ± 34.7; DCP: 28.4 ± 22.0; *P* = 0.004), indicating more preserved speech and swallowing function in general.

**Figure 2:**
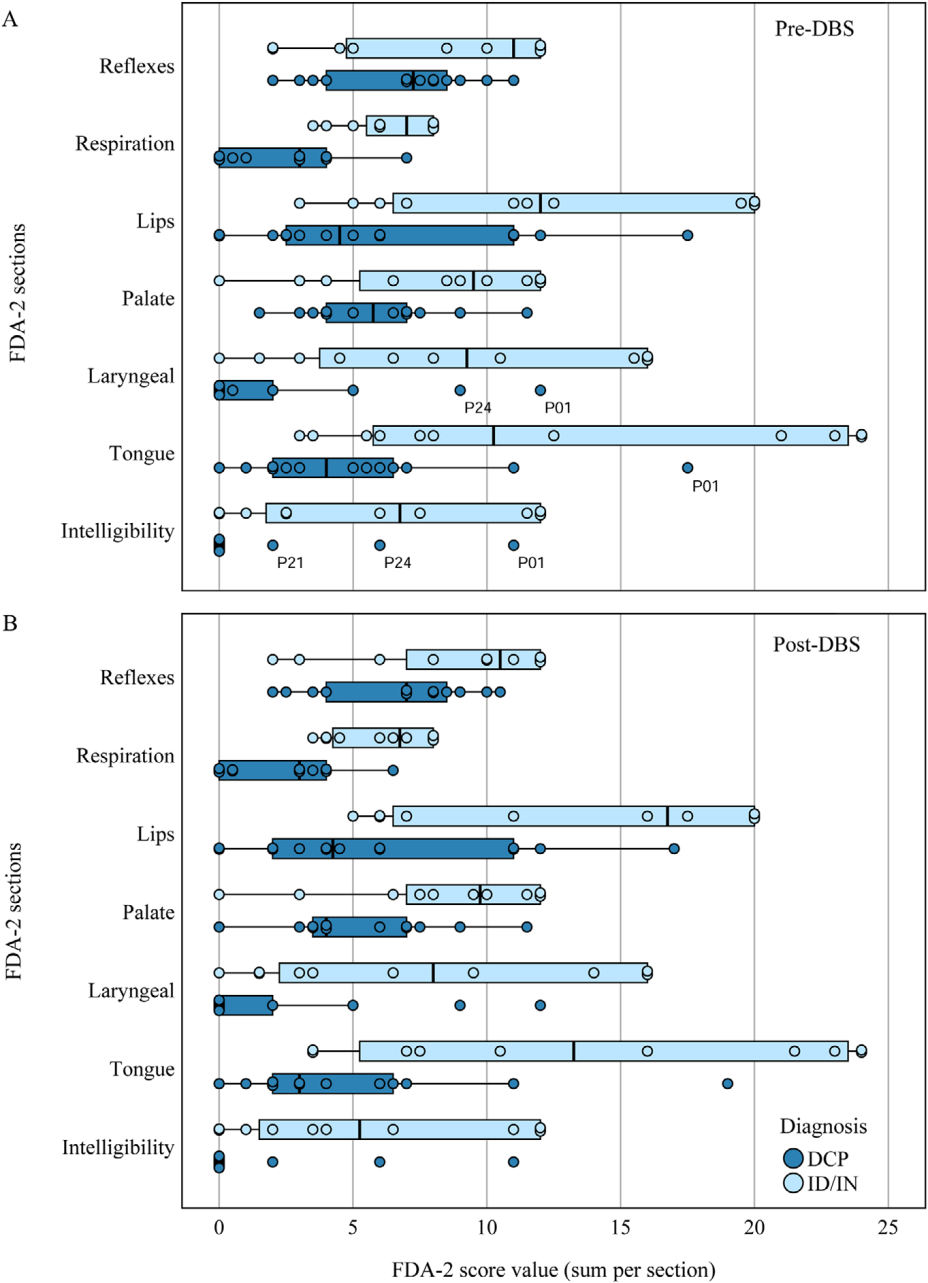
FDA‐2 section scores before surgery (pre) and 12 months after surgery (post) in patients with DCP (blue) or ID/IN (light blue). Individual measurements combined with box plots, which display first and third quartile (bottom and top line of the box), median (middle line), whiskers show maximum and minimum values with the exceptions of outliers (circles, distance more than 1.5 times of IQR) and extremes (asterisks, distance more than three times of IQR).

Figure 2 depicts the results for individual sections by group. Pre‐DBS, the most pronounced differences were observed in respiration (ID/IN: 6.5 ± 1.7; DCP: 2.3 ± 2.1; *P* < 0.001), lips (ID/IN: 13.0 ± 6.7; DCP: 5.9 ± 5.1; *P* = 0.007), laryngeal function (ID/IN: 9.5 ± 6.3; DCP: 2.0 ± 3.9; *P* = 0.002), and intelligibility (ID/IN: 6.6 ± 5.2; DCP: 1.4 ± 3.2; *P* = 0.007), with ID/IN patients consistently scoring higher. These differences persisted post‐surgery. However, the magnitude of change following DBS did not differ significantly between groups for any section (Supplement Table 1b, Supplement Table 1c).

Although not reaching statistical significance, ID/IN patients showed a trend towards improvements in the sections’ reflexes (change: 0.5 ± 1.1, *P* = 0.132), lips (change: 1.1 ± 3.9, *P* = 0.360), and tongue (change: 0.5 ± 1.9, *P* = 0.389).

Categorization of FDA‐2 items into three functional domains (oral structure at rest, nonverbal oromotor function, and motor speech function) confirmed significant differences between ID/IN and DCP groups across all categories both pre‐ and post‐DBS (*P* < 0.05), with ID patients consistently scoring higher.

The nonverbal oromotor function category showed the largest mean improvement, particularly for the ID/IN group (1.2 ± 5.4, *P* = 0.456), although the change as well as the between‐group difference in change was not statistically significant (ID/IN − DCP: 2.0 ± 1.6, *P* = 0.246). The motor speech function category demonstrated minimal change in both groups (Supplement Tables 1b and 1c).

Analysis of estimated marginal means (EMMs), adjusted for age, antidystonic medication, and pre‐surgical values, did not reveal a notable reduction in the differences between ID/IN and DCP groups post‐surgery. In fact, the FDA‐2 total score difference slightly increased (ID/IN: 47.5 [43.8–51.2], DCP: 45.3 [41.9–48.6]; difference: 2.2, *P* = 0.407), as did the difference in nonverbal oromotor function (ID/IN: 29.1 [26.4–31.7], DCP: 26.5 [24.1–28.8]; difference: 2.6, *P* = 0.170). The difference between the two groups narrowed only in oral structure at rest (ID/IN: 8.0 [7.6–8.4], DCP: 7.7 [7.3–8.1]; difference: 0.3, *P* = 0.401). Interestingly, adjusted post‐surgery scores for motor speech function showed a reversal of the previous trend, with DCP patients scoring slightly higher than ID/IN patients (DCP: 11.1 [10.3–11.9], ID/IN: 10.4 [9.5–11.3]; difference: −0.7, *P* = 0.273). This reversal reflects statistical adjustment for the substantial baseline differences between groups (DCP: 4.4 ± 8.7; ID/IN: 18.8 ± 12.7), suggesting that relative to their starting points, DCP patients retained motor speech function proportionally better than ID/IN patients, although these differences remained small and non‐significant.

Changes from pre‐surgery, when adjusted for covariates, were minimal and not statistically significant for all categories. Detailed results are presented in Table 2.

**TABLE 2:** FDA‐2 total score and category scores before surgery (pre‐DBS) and 12 months after surgery (post‐DBS) in patients with DCP and ID/IN

	Descriptive summary^a^				EMMs^b^	
	Pre‐DBS	Post‐DBS	Change (post–pre)	*P*‐value (post–pre)	Post‐DBS	Change from Pre‐DBS
FDA‐2 total						
ID/IN	66.1 (35.0)	67.1 (34.7)	1.0 (7.5)	0.641	47.5 [43.8 to 51.2]^c^	1.2 [‘‐2.5 to 4.9]^c^, *P* = 0.495
DCP	29.3 (21.6)	28.4 (22.0)	−0.8 (1.4)	0.046	45.3 [41.9 to 48.6]^c^	−0.9 [−4.3 to 2.3]^c^, *P* = 0.549
Difference	36.8	38.7	1.9		2.2	
95%‐CI	[12.4 to 61.3]	[14.3 to 63.1]	[−3.0 to 6.7]		[−3.2 to 7.6]	
*P*‐value	0.005	0.004	0.415		0.407	
Oral structure (at rest)						
ID/IN	10.0 (1.7)	10.4 (1.6)	0.3 (0.9)	0.207	8.0 [7.6 to 8.4]^d^	0.3 [−0.2 to 0.7]^d^, *P* = 0.195
DCP	5.8 (3.8)	5.7 (3.9)	0.0 (0.4)	0.720	7.7 [7.3 to 8.1]^d^	0.0 [−0.4 to 0.4]^d^, *P* = 0.959
Difference	4.3	4.7	0.4		0.3	
95%‐CI	[1.9 to 6.6]	[2.3 to 7.0]	[−0.2 to 0.9]		[−0.4 to 0.9]	
*P*‐value	0.001	0.001	0.188		0.401	
Nonverbal oromotor function						
ID/IN	37.3 (21.0)	38.5 (2.2)	1.2 (5.4)	0.456	29.1 [26.4 to 31.7]^e^	1.6 [−1.1 to 4.2]^e^, *P* = 0.229
DCP	19.1 (10.9)	18.4 (11.5)	−0.8 (1.4)	0.068	26.5 [24.1 to 28.8]^e^	−1.0 [−3.4 to 1.3]^e^, *P* = 0.371
Difference	18.2	20.1	2.0		2.6	
95%‐CI	[3.9 to 32.5]	[6.2 to 34.0]	[−1.5 to 5.5]		[−1.2 to 6.4]	
*P*‐value	0.016	0.007	0.246		0.170	
Motor speech function						
ID/IN	18.8 (12.7)	18.3 (13.1)	−0.5 (1.8)	0.349	10.4 [9.5 to 11.3]^f^	−0.6 [−1.5 to 0.2]^f^, *P* = 0.147
DCP	4.4 (8.7)	4.4 (8.6)	0.0 (0.1)	0.336	11.1 [10.3 to 11.9]^f^	0.1 [−0.7 to 0.8]^f^, *P* = 0.862
Difference	14.4	13.9	0.5		−0.7	
95%‐CI	[5.3 to 23.4]	[4.6 to 23.2]	[−1.6 to 0.7]		[−1.9 to 0.6]	
*P*‐value	0.004	0.006	0.384		0.273	

Abbreviations: ANOVA, analysis of variance; CI, confidence interval; EMM, estimated marginal mean.

^a^ Descriptive results are given as mean (standard deviation); differences between groups are calculated as ID/IN minus DCP, 95% CI and *P*‐values derived from t‐test (paired or unpaired, 2‐sided, respectively), data collection was complete for all patients (ID/IN: *n* = 12, DCP: *n* = 14).

^b^ EMM (95% CI and *P*‐values) were derived from ANOVA outcome: post‐surgical score or change from pre‐surgery, adjusted for age, antidyston, medication and pre‐surgical value (no interaction).

^c^ Covariates appearing in the model were evaluated at the following values: Age at DBS = 12.15 (for all), pre‐surgery values of the score or the respective category: 46.250 (total).

^d^ 7.731 (oral structure).

^e^ 27.500 (nonverbal motor function).

^f^ 11.019 (motor speech function).

**Figure 3:**
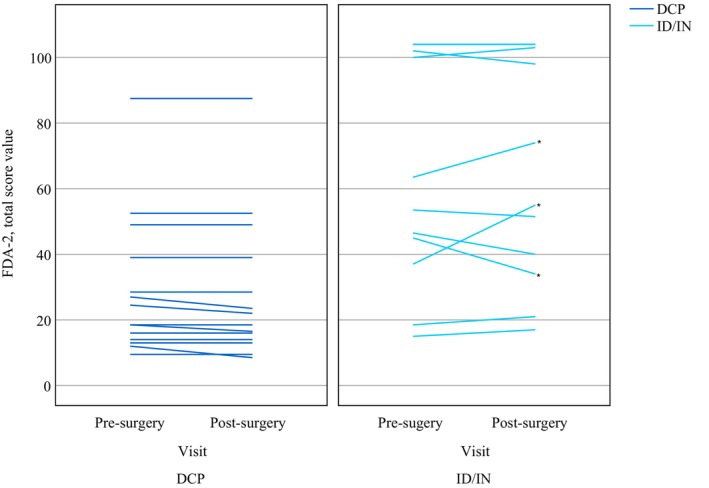
Course of individual FDA‐2 total scores in patients with DCP (left, blue) and ID/IN (right, light blue). Three patients showed noteworthy changes: a 14‐yo female (KMT2B) improved from 37 to 55 (+18; +3 reflexes, +11.5 lip control, +4.5 tongue function). A 16‐yo female (ADCY5) improved from 63.5 to 74 (+10.5; +3.5 lip control, +3.5 tongue function). A 16‐yo female (PANK2) declined from 45 to 34 (−11; −7.5 laryngeal, −5.5 lip control).

Notable individual responses were observed exclusively in the ID/IN group, with patient P13 (KMT2B) showing marked improvement in FDA‐2 total score (+18 points), patient P14 (ADCY5) demonstrating moderate improvement (+10.5 points), and patient P16 (PANK2) experiencing deterioration (−11 points), as detailed in Figure 3.

**Figure 4:**
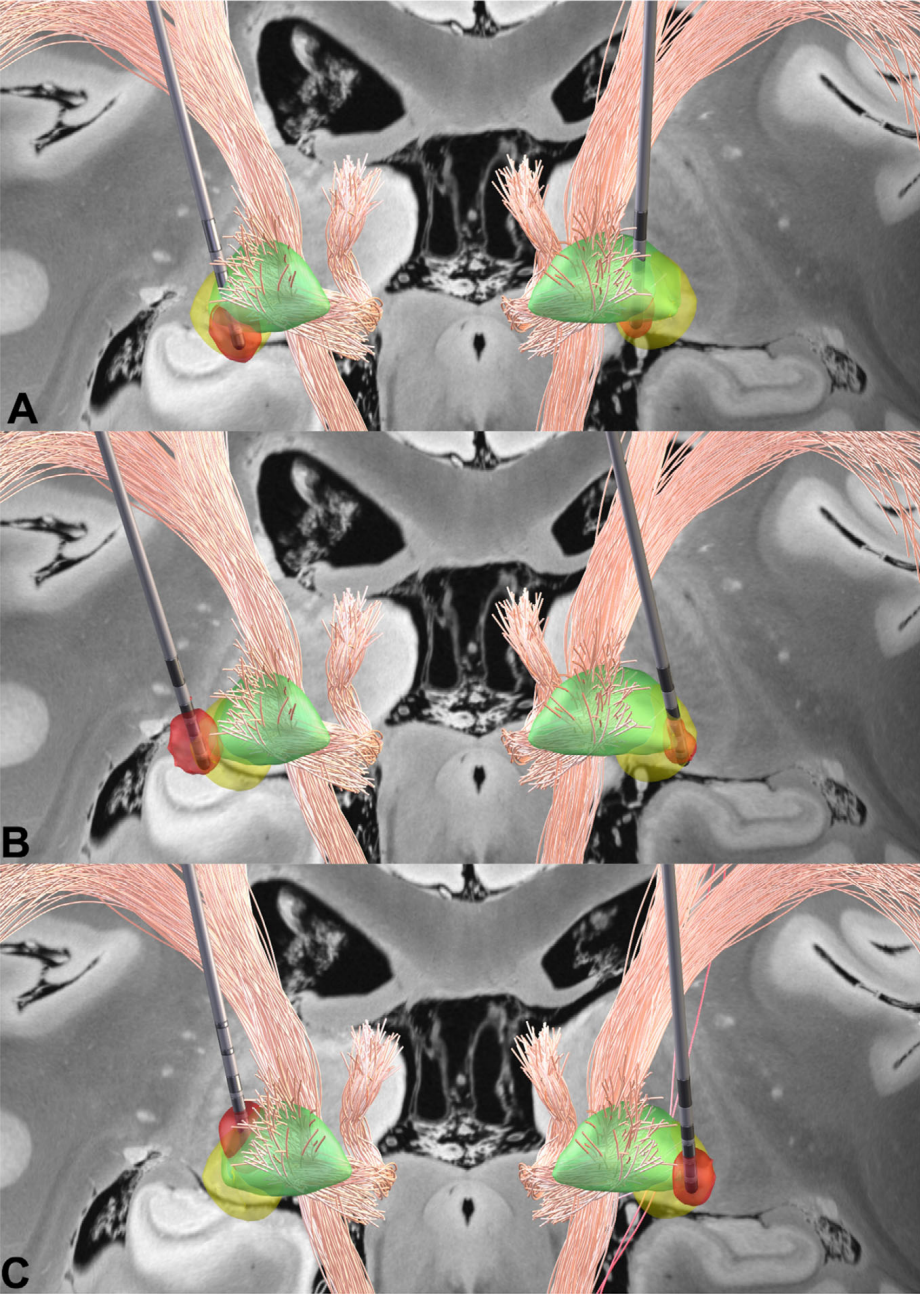
Lead‐DBS v3^27^ visualization of electrode placement in three patients with generalized dystonia. The internal globus pallidus (GPi) is highlighted in green, with the Volume of Tissue Activated (VTA) shown in red and sweet spot tracts in yellow, indicating regions positively correlated with symptom improvement. (A) Patient 13: Bilateral stimulation parameters – C (+); 1 (−) 30%, 2–4 (−) 70%; 3.4 mA, 120 μs, 130 Hz. (B) Patient 14: Left – C (+); 2(−) 45%, 3 (−) 15%, 5(−) 30%, 6(−) 10%; 2.5 mA, 60 μs, 159 Hz. Right – C (+); 10 (−) 70%, 13 (−) 30%; 3.5 mA, 60 μs, 159 Hz. (C) Patient 16: Bilateral stimulation parameters – C (+); 1 (−) 50%, 2–4 (−) 50%; 3.3 mA, 120 μs, 130 Hz.

Figure 4 shows Lead‐DBS v3^28^ visualization of electrode placement and optimal stimulation achievement in patients P13, P14 and P16. Individual stimulation settings of all patients are reported in Supplementary Table 1d.

## Discussion

This study investigated the effects of GPi‐DBS on speech and swallowing functions in pediatric patients with dystonia, comparing outcomes according to the etiology of ID/IN and DCP.

A notable finding was the substantial baseline variability observed in FDA‐2 scores. This extensive variability likely reflects the inherent heterogeneity within pediatric dystonia populations, and may be attributed to the diverse etiologies, severity levels, and individual patient characteristics within our cohort, particularly given our inclusion of both acquired (DCP) and inherited dystonias (ID/IN).

A key finding of our study is the consistently higher FDA‐2 scores in ID/IN patients compared to DCP patients at baseline. This difference reflects fundamental distinctions in the underlying pathophysiology and clinical presentations between these patient populations.

The categorization of FDA‐2 items into distinct functional domains (oral structure at rest, nonverbal oromotor function, and motor speech function)^26^ confirmed these significant baseline differences across all categories, with ID/IN patients consistently demonstrating superior performance.

Our results indicate that GPi‐DBS did not lead to significant overall changes in speech and swallowing functions, as measured by the FDA‐2 total scores and individual subitems across the entire cohort. This finding is consistent with some previous studies in adult populations that reported variable or minimal effects of DBS on speech and swallowing.^17^

The magnitude of change following DBS did not differ significantly between ID/IN and DCP groups for any category, suggesting a limited therapeutic response regardless of dystonia etiology. Nevertheless, the observation that nonverbal oromotor function showed the largest mean improvement in ID/IN patients, albeit non‐significant trends, suggests that GPi‐DBS may have a more pronounced effect on general oromotor control than on specific speech production.^29^ Similarly, small, non‐significant positive trends were observed in ID/IN patients in lips and tongue sections post‐DBS. This trend needs to be further investigated in larger cohorts.

When controlling for potential confounding factors through estimated marginal means analysis (adjusted for age, antidystonic medication, and pre‐surgical values), the group differences persisted post‐surgery, indicating that the baseline disparities between ID/IN and DCP patients were not substantially altered by DBS intervention. Notably, the adjusted analysis revealed that DCP patients demonstrated a slightly better relative outcome in motor speech function compared to ID/IN patients when accounting for their baseline scores, despite starting from significantly lower absolute values.

Examining individual patient responses reveals a various and heterogeneous picture of outcomes, highlighting the complexity of dystonia as a disorder, particularly in pediatric populations.^30^ We observed greater variability in the ID/IN group, with some notable improvements in individual patients, such as P13 and P14, or evident deterioration, as in patient P16. Of particular interest, patient P13, who carries the KMT2B mutation, a condition typically characterized by substantial oromotor involvement, articulation difficulties and reduced speech volume,^31^ showed positive changes in several FDA‐2 items following GPi‐DBS, especially in reflexes, lip control, and tongue function, aligning with previously reported data on dysarthria improvement after DBS in patients carrying KMT2B variants.^32^, ^33^, ^34^

Patient P14, with a pathogenic ADCY5 gene mutation, showed moderate improvement primarily in lip and tongue function. ADCY5‐related disorders often present with complex hyperkinetic movement affecting multiple body regions, including prominent orofacial dyskinesia.^35^ The specific improvement in oromotor control suggests potential benefit of GPi‐DBS for speech‐related symptoms in this genetic condition, although data on ADCY5 patients undergoing DBS remain limited with only few case reports available to date.^36^, ^37^

Conversely, the deterioration observed in patient P16, who harbors a PANK2 pathogenic mutation, particularly in laryngeal function and lip control, might be attributed, at least partially, to the natural progression of the disease over time.^38^

Our study may have been limited by the relatively small sample size and the heterogeneity within the ID/IN and DCP groups emphasizing the need for further investigation with larger sample sizes and longer follow‐up periods. Combining retrospective and prospective data from different sources (STIM‐CP trial and GEPESTIM registry) may have introduced bias in the original patient selection and timing for DBS procedures, contributing to the overall sample heterogeneity. To mitigate these potential biases, we standardized outcome measures and assessment timepoints during analysis.

Stimulation parameters, electrode position, and underlying genetic mutations may have influenced the observed outcomes, making it challenging to establish direct correlations between specific stimulation parameters and FDA‐2 outcomes. Moreover, the stimulation settings were optimized primarily to reduce tone and hyperkinesia on neck, trunk and limbs in order to improve motor function, rather than being adjusted specifically to improve speech and swallowing, which may have limited potential benefits in these domains. Larger patient cohorts will be necessary to better understand the effects of DBS on oromotor function and speech in specific monogenic dystonias and to identify potential predictors of treatment response.

Evaluating speech and swallowing in pediatric patients with dystonia, especially those with impaired expressive language and/or intellectual disability, presents significant challenges. While the FDA‐2 offers several advantages, including its task‐based nature and ability to provide information on both speech and swallowing when administered by an experienced speech therapist, it may not fully capture the nuances of communication in these severely affected children. The FDA‐2, was originally designed and validated for individuals aged 12 years and older, and extending its use to younger children in our cohort, might have affected assessment reliability. Additionally, the FDA‐2 does not account for nonverbal communication, which may be a primary mode of interaction for some patients in our cohort, and contains inherent bias when assessing verbal versus non‐verbal children, as non‐verbal patients cannot complete the “intelligibility” section, systematically resulting in lower overall scores. While the FDA‐2 is widely validated with robust psychometric properties demonstrated across various linguistic‐cultural adaptations, a reference value for minimally clinically significant change remains unestablished. To interpret score changes in our study, we relied on normative data and test–retest reliability values from the original manual and validation studies.

Other existing assessment tools, scales such as the Viking Speech Scale,^39^ the Communication Function Classification System (CFCS)^27^ for speech, and the Eating and Drinking Ability Classification System (EDACS)^40^ or the Sydney Swallowing Questionnaire^41^ for swallowing, offer broader functional classifications but provide less detailed assessment than the FDA‐2.

The Bogenhausen Dysarthria Scales (BoDyS)^42^ represents another dysarthria assessment tool originally designed for adults that has been applied to evaluate dysarthria in children with neurological conditions,^43^, ^44^ though the FDA‐2's advantage lies in its comprehensive assessment of non‐speech oral motor functions.

For adult populations with laryngeal dystonia, additional subjective assessments like the Voice Handicap Index (VHI)^45^ and the Vocal Performance Questionnaire (VPQ)^46^ and objective acoustic analysis tools such as the Acoustic Voice Quality Index (AVQI),^47^ the Cepstral Spectral Index of Dysphonia (CSID),^48^, ^49^ and the Cepstral Peak Prominence Smoothed (CPPS)^49^, ^50^ are available, though these may have limited applicability in analyzing the complex speech disorders observed in our severely affected pediatric patients who may present with concurrent laryngeal and orofacial dystonia, as well as dysphagia and global developmental delay.

In conclusion, this study provides valuable insights into the effects of GPi‐DBS on speech and swallowing functions in pediatric patients with dystonia. While overall FDA‐2 scores remained stable following GPi‐DBS, individual patient improvements and the differences observed between ID/IN and DCP groups underscore the complex nature of treatment responses, highlighting the need for personalized management strategies. Therefore, future research should integrate more complex and patient‐centered assessment tools to fully assess the effects of GPi‐DBS on domains like speech and swallowing that might not be reflected in detailed FDA‐2 scores. The analysis of imaging data to gain more insights into the topographical organization of the GPi including connectivity analysis, or even the exploration of alternative targets with individualized testing protocols, could substantially contribute to our understanding of DBS effects across different dystonia etiologies in pediatric populations and to improve DBS treatment in children with dystonia.

Integrating aspects of broader functional classification scales could provide a more comprehensive view of overall functional communication and swallowing abilities that might not be reflected in detailed FDA‐2 scores.^51^

## Author Roles

(1) Research project: A. Conception, B. Organization, C. Execution; (2) Statistical Analysis: A. Design, B. Execution, C. Review and Critique; (3) Manuscript: A. Writing of the first draft, B. Review and Critique.

K.B.: 1A, 1B, 1C, 3A.

A.A.K.: 3B.

A.L.A.M.: 3B.

M.E.: 3B.

C.R.Z.: 3B.

J.K.K.: 3B.

J.R.: 3B.

R.M.F.: 1C, 3B.

D.L.: 3B.

M.M.: 3B.

A.S.: 3B.

A.B.: 3B.

L.v.S.: 3B.

V.V.V.: 3B.

L.T.: 3B.

P.S.: 2A, 2B, 2C, 3B.

A.K.: 1A, 1B, 3B.

## Disclosures

**Ethical Compliance Statement:** This study analyzes data from the STIM‐CP trial (NCT02097693) and the GEPESTIM registry (DRKS813‐168), both approved by the ethics committee of the University of Cologne and by the local ethics committees of the participating centers. All capable patients or their legal caregivers provided written informed consent. We confirm that we have read the Journal's position on issues involved in ethical publication and affirm that this work is consistent with those guidelines.

**Funding Sources and Conflict of Interest:** Funding Agencies: STIM‐CP was partly funded by Boston Scientific Neuromodulation, Valencia, USA, who had no influence on study design or conduct, data collection or interpretation, clinical management of patients, or writing of the report. Further funding sources were the University of Cologne and the Dr. Hans Günther and Dr. Rita Herfort Foundations. AAK and ALAM are supported by the Collaborative Research Centre TRR 295 (Project ID 4247788381) of the “Deutsche Forschungsgemeinschaft” (DFG). AAK is supported by DFG under Germany's Excellence Strategy EXC‐2049 – 390,688,087 and additionally funded by the Lundbeck Foundation (Grant Nr. R336‐2020‐1035). The corresponding author and writing committee had full access to the data and had final responsibility for the decision to submit the paper for publication. Relevant Conflicts of Interest/Financial Disclosures: AK reports grants from Dr. Hans Günther and Dr. Rita Herfort Foundation. The institution of AK, not AK personally received funding by Boston Scientific Neuromodulation. AAK has served on advisory boards of Medtronic and has received honoraria and travel support from Medtronic and Boston Scientific outside of this work. JKK is a consultant to Medtronic and Boston Scientific.

**Financial Disclosures for the previous 12 months:** The authors declare that there are no additional disclosures to report.

## Supporting information


**TABLE S1a.** Etiology and Communication Function in DCP patients
**TABLE S1b.** FDA‐2 total, sections and category scores for patients with DCP and ID/IN before surgery
**TABLE S1c.** FDA‐2 total, sections and category scores for patients with DCP and ID/IN 12 months after surgery
**TABLE S1d.** Gpi‐BDS Stimulation Parameters of the included Patients.

## Data Availability

The data that support the findings of this study are available from the corresponding author upon reasonable request.

## References

[1] Albanese A , Bhatia KP , Fung VSC , et al. Definition and classification of dystonia. Mov Disord 2025;40(7):1248–1259. 10.1002/mds.30220. 40326714 PMC12273609

[2] Meijer IA , Pearson TS . The twists of pediatric dystonia: phenomenology, classification, and genetics. Semin Pediatr Neurol 2018;25:65–74. 29735118 10.1016/j.spen.2018.02.001

[3] Fehlings DL , Zarrei M , Engchuan W , Sondheimer N , Thiruvahindrapuram B , MacDonald JR , et al. Comprehensive whole‐genome sequence analyses provide insights into the genomic architecture of cerebral palsy. Nat Genet 2024;56(4):585–594. 38553553 10.1038/s41588-024-01686-x

[4] Koy A , Pauls KAM , Flossdorf P , et al. Young adults with dyskinetic cerebral palsy improve subjectively on pallidal stimulation, but not in formal dystonia, gait, speech and swallowing testing. Eur Neurol 2014;72(5–6):340–348. 25322688 10.1159/000360984

[5] Monbaliu E , Himmelmann K , Lin JP , et al. Clinical presentation and management of dyskinetic cerebral palsy. Lancet Neurol 2017;16:741–749. 28816119 10.1016/S1474-4422(17)30252-1

[6] Koy A , Kühn AA , Huebl J , et al. Quality of life after deep brain stimulation of pediatric patients with dyskinetic cerebral palsy: a prospective, single‐arm, multicenter study with a subsequent randomized double‐blind crossover (STIM‐CP). Mov Disord 2022;37(4):799–811. 34967053 10.1002/mds.28898

[7] Klein C . Genetics in dystonia. Parkinsonism Relat Disord 2014;20(S1):S137–S142. 24262166 10.1016/S1353-8020(13)70033-6

[8] Koy A , Lin JP , Sanger TD , Marks WA , Mink JW , Timmermann L . Advances in management of movement disorders in children. Lancet Neurol 2016;15:719–735. 27302239 10.1016/S1474-4422(16)00132-0

[9] Roubertie A , Mariani LL , Fernandez‐Alvarez E , Doummar D , Roze E . Treatment for dystonia in childhood. Eur J Neurol 2012;19:1292–1299. 22289078 10.1111/j.1468-1331.2011.03649.x

[10] Volkmann J , Wolters A , Kupsch A , et al. Pallidal deep brain stimulation in patients with primary generalised or segmental dystonia: 5‐year follow‐up of a randomised trial. Lancet Neurol 2012;11(12):1029–1038. 23123071 10.1016/S1474-4422(12)70257-0

[11] Kupsch A , Benecke R , Müller J , Trottenberg T , Schneider GH , Poewe W , et al. Pallidal deep‐brain stimulation in primary generalized or segmental dystonia. N Engl J Med 2006;355(19):1978–1990. 17093249 10.1056/NEJMoa063618

[12] Vidailhet M , Vercueil L , Houeto JL , Krystkowiak P , Benabid AL , Cornu P , et al. Bilateral deep‐brain stimulation of the globus pallidus in primary generalized dystonia. N Engl J Med 2005;352(5):459–467. 15689584 10.1056/NEJMoa042187

[13] Lumsden DE , Kaminska M , Gimeno H , et al. Proportion of life lived with dystonia inversely correlates with response to pallidal deep brain stimulation in both primary and secondary childhood dystonia. Dev Med Child Neurol 2013;55(6):567–574. 23452222 10.1111/dmcn.12117

[14] Elia AE , Bagella CF , Ferré F , Zorzi G , Calandrella D , Romito LM . Deep brain stimulation for dystonia due to cerebral palsy: a review. Eur J Paediatr Neurol 2018;22:308–315. 29396170 10.1016/j.ejpn.2017.12.002

[15] Marks WA , Honeycutt J , Acosta F , Reed M , Bailey L , Pomykal A , et al. Dystonia due to cerebral palsy responds to deep brain stimulation of the globus pallidus internus. Mov Disord 2011;26(9):1748–1751. 21491490 10.1002/mds.23723

[16] Austin A , Lin JP , Selway R , Ashkan K , Owen T . What parents think and feel about deep brain stimulation in paediatric secondary dystonia including cerebral palsy: a qualitative study of parental decision‐making. Eur J Paediatr Neurol 2017;21(1):185–192. 27658770 10.1016/j.ejpn.2016.08.011

[17] Baudouin R , Lechien JR , Carpentier L , Gurruchaga JM , Lisan Q , Hans S . Deep brain stimulation impact on voice and speech quality in Parkinson's disease: a systematic review. Otolaryngology 2023;168(3):307–318. 10.1177/01945998221120189 36040825

[18] Finger ME , Siddiqui MS , Morris AK , Ruckart KW , Wright SC , Haq IU , Madden LL . Auditory‐perceptual evaluation of deep brain stimulation on voice and speech in patients with dystonia. J Voice 2020;34(4):636–644. 30879706 10.1016/j.jvoice.2019.02.010 PMC6745002

[19] Henry KA , Singh R , Zhang N , Lyons MK , McNett K , Neal MT , et al. Effect of STN/GPi DBS on swallowing function in Parkinson's disease as assessed by video fluoroscopy: a retrospective study. Parkinsonism Relat Disord 2022;103:136–140. 36115199 10.1016/j.parkreldis.2022.08.017

[20] Vidailhet M , Vercueil L , Houeto JL , Krystkowiak P , Lagrange C , Yelnik J , et al. Bilateral, pallidal, deep‐brain stimulation in primary generalised dystonia: a prospective 3 year follow‐up study; Available from: http://neurology.thelancet.com. 10.1016/S1474-4422(07)70035-2 17303528

[21] Risch V , Staiger A , Ziegler W , Ott K , Schölderle T , Pelykh O , et al. How does GPi‐DBS affect speech in primary dystonia? Brain Stimul 2015;8(5):875–880. Available from: http://www.brainstimjrnl.com/article/S1935861X1500933X/fulltext. 26002621 10.1016/j.brs.2015.04.009

[22] Enderby P . Frenchay dysarthria assessment. Int J Lang Commun Disord 1980;15(3):165–173.

[23] Pawlukowska W , Szylińska A , Kotlęga D , Rotter I , Nowacki P . Differences between subjective and objective assessment of speech deficiency in Parkinson disease. J Voice 2018;32(6):715–722. 29122413 10.1016/j.jvoice.2017.08.018

[24] Keage MJ , Delatycki MB , Gupta I , Corben LA , Vogel AP . Dysphagia in Friedreich Ataxia. Dysphagia 2017;32(5):626–635. 28474131 10.1007/s00455-017-9804-4

[25] Hijikata N , Kawakami M , Wada A , et al. Assessment of dysarthria with Frenchay dysarthria assessment (FDA‐2) in patients with Duchenne muscular dystrophy. Disabil Rehabil 2022;44(8):1443–1450. 32772581 10.1080/09638288.2020.1800108

[26] Cardoso R , Guimarães I , Santos H , et al. Frenchay dysarthria assessment (FDA‐2) in Parkinson's disease: cross‐cultural adaptation and psychometric properties of the European Portuguese version. J Neurol 2017;264(1):21–31. 27747392 10.1007/s00415-016-8298-6

[27] Cunningham BJ , Rosenbaum P , Hidecker MJC . Promoting consistent use of the communication function classification system (CFCS). Disabil Rehabil 2016;38(2):195–204. 25801921 10.3109/09638288.2015.1027009

[28] Neudorfer C , Butenko K , Oxenford S , Rajamani N , Achtzehn J , Goede L , et al. Lead‐DBS v3.0: mapping deep brain stimulation effects to local anatomy and global networks. NeuroImage 2023;268:119862. 36610682 10.1016/j.neuroimage.2023.119862 PMC10144063

[29] Flamand‐Rouvière C , Guettard E , Moreau C , et al. Speech disturbances in patients with dystonia or chorea due to neurometabolic disorders. Mov Disord 2010;25(11):1605–1611. 20629163 10.1002/mds.23134

[30] Fernández‐Alvarez E . Dystonia. The paediatric perspective. Eur J Neurol 2010;17:46–51. 20590808 10.1111/j.1468-1331.2010.03050.x

[31] Abela L , Kurian MA . KMT2B‐Related Dystonia. GeneReviews® [Internet]; 2022. [cited 2025 Feb 10]; Available from: https://www.ncbi.nlm.nih.gov/books/NBK493766/.

[32] Abel M , Pfister R , Hussein I , Alsalloum F , Onyinzo C , Kappl S , et al. Deep brain stimulation in KMT2B‐related dystonia: case report and review of the literature with special emphasis on dysarthria and speech. Front Neurol 2021;12:662910. 34054706 10.3389/fneur.2021.662910 PMC8160374

[33] Cif L , Demailly D , Lin JP , Barwick KE , Sa M , Abela L , et al. KMT2B‐related disorders: expansion of the phenotypic spectrum and long‐term efficacy of deep brain stimulation. Brain 2020;143(11):3242–3261. 33150406 10.1093/brain/awaa304 PMC7719027

[34] Zech M , Lam DD , Winkelmann J . Update on KMT2B‐related dystonia. Curr Neurol Neurosci Rep 2019;19(11):92. 31768667 10.1007/s11910-019-1007-y

[35] Yang K , Ebrahimi‐Fakhari D . *ADCY5*‐related movement disorder. In: Adam MP , Feldman J , Mirzaa GM , Pagon RA , Wallace SE , Amemiya A , eds. GeneReviews. Seattle (WA): University of Washington, Seattle; 2014. 25521004

[36] de Almeida Marcelino AL , Mainka T , Krause P , Poewe W , Ganos C , Kühn AA . Deep brain stimulation reduces (nocturnal) dyskinetic exacerbations in patients with ADCY5 mutation: a case series. J Neurol 2020;267(12):3624–3631. 10.1007/s00415-020-09871-8. 32647899 PMC7674568

[37] Cif L , Demailly D , Gehin C , et al. Deep brain stimulation effect in genetic dyskinetic cerebral palsy: the case of ADCY5‐related disease. Mol Genet Metab 2023;138(1):106970. 10.1016/j.ymgme.2022.106970. 36610259

[38] Hayflick SJ , Kurian MA , Hogarth P . Neurodegeneration with brain iron accumulation. Handb Clin Neurol 2018;147:293. 29325618 10.1016/B978-0-444-63233-3.00019-1 PMC8235601

[39] Pennington L , Hustad KC . Construct validity of the Viking speech scale. Folia Phoniatr Logop 2019;71(5–6):228–237. 31189170 10.1159/000499926 PMC7110417

[40] Bykova KM , Frank U , Girolami GL . Eating and drinking ability classification system to detect aspiration risk in children with cerebral palsy: a validation study. Eur J Pediatr 2023;182(7):3365–3373. 37184644 10.1007/s00431-023-04998-y PMC10183305

[41] Búa BA , Bülow M . Validation in Swedish of Sydney swallow questionnaire. BMC Res Notes 2014;7(1):742. 25330714 10.1186/1756-0500-7-742 PMC4216845

[42] Ziegler W , Staiger A , Schölderle T , Vogel M . Gauging the auditory dimensions of dysarthric impairment: reliability and construct validity of the Bogenhausen dysarthria scales (BoDyS). J Speech Lang Hear Res 2017;60(6):1516–1534. 28538944 10.1044/2017_JSLHR-S-16-0336

[43] Schölderle T , Haas E , Ziegler W . Dysarthria syndromes in children with cerebral palsy. Dev Med Child Neurol 2021;63(4):444–449. 32970343 10.1111/dmcn.14679

[44] Schölderle T , Haas E , Ziegler W . Speech Naturalness in the Assessment of Childhood Dysarthria. Am J Speech Lang Pathol 2023;32(4):1633–1643. Available from: 10.1044/2023_AJSLP-23-00023?url_ver=Z39.88-2003&rfr_id=ori:rid:crossref.org&rfr_dat=cr_pub++0pubmed. 37343549

[45] Jacobson BH , Johnson A , Grywalski C , Silbergleit A , Jacobson G , Benninger MS , et al. The voice handicap index (VHI). Am J Speech Lang Pathol 1997;6(3):66–69.

[46] Carding PN , Horsley IA , Docherty GJ . A study of the effectiveness of voice therapy in the treatment of 45 patients with nonorganic dysphonia. J Voice 1999;13(1):72–104. 10223677 10.1016/s0892-1997(99)80063-0

[47] Maryn Y , Corthals P , Van Cauwenberge P , Roy N , De Bodt M . Toward improved ecological validity in the acoustic measurement of overall voice quality: combining continuous speech and sustained vowels. J Voice 2010;24(5):540–555. 19883993 10.1016/j.jvoice.2008.12.014

[48] Awan SN , Roy N , Dromey C . Estimating dysphonia severity in continuous speech: application of a multi‐parameter spectral/cepstral model. Clin Linguist Phon 2009;23(11):825–841. 19891523 10.3109/02699200903242988

[49] Esen Aydinli F , Özcebe E , İncebay Ö . Use of cepstral analysis for differentiating dysphonic from normal voices in children. Int J Pediatr Otorhinolaryngol 2019;116:107–113. 30554679 10.1016/j.ijporl.2018.10.029

[50] Dwyer CD , Gochman GE , Rosen CA , Young VVN , Schneider SL . Comparison of outcome measures (subjective, objective, and patient‐based) in laryngeal dystonia treatment with botulinum toxin a injection. J Voice 2023;39(5):1302–1312. 37121839 10.1016/j.jvoice.2023.03.019

[51] Liker MA , Sanger TD , MacLean JA , et al. Stereotactic awake basal ganglia electrophysiological recording and stimulation (SABERS): a novel staged procedure for personalized targeting of deep brain stimulation in pediatric movement and neuropsychiatric disorders. J Child Neurol 2024;39(1–2):33–44. 10.1177/08830738231224057. 38409793

