# Supplementary material for: Effects of Pallidal Deep Brain Stimulation on Speech and Swallowing in Pediatric Patients with Dystonia
Source: Mov Disord Clin Pract. 2025 Nov 27;13(5):1194–205. doi: 10.1002/mdc3.70454 (PMC13172749; doi:10.1002/mdc3.70454)
Supplement: Supplementary file 1 — TABLE S1a. Etiology and Communication Function in DCP patients TABLE S1b. FDA‐2 total, sections and category scores for patients with DCP and ID/IN before surgery TABLE S1c. FDA‐2 total, sections and category scores for patients with DCP and ID/IN 12 months after surgery TABLE S1d. Gpi‐BDS Stimulation Parameters of the included Patients. [file MDC3-13-1194-s001.docx]

**Supplementary Table 1a.** Etiology and Communication Function in DCP patients.

| **ID** | **DCP Etiology** | **Brain Lesions** | **APGAR** | **CSCF Score** |
| --- | --- | --- | --- | --- |
| P01 | HIE, Perinatal Stroke, Prematurity (36 weeks) | Basal ganglia, Thalamus, Grey matter | 4-6-8 | II |
| P02 | HIE | Basal ganglia, Thalamus | 0-6-7 | IV |
| P03 | HIE | Basal ganglia, Thalamus | 0-7-7 | V |
| P04 | HIE, Prematurity (31 weeks) | Basal ganglia, Thalamus | 1-1-3 | V |
| P11 | HIE | Basal ganglia, Temporo-polar atrophy | Missing | V |
| P17 | HIE | Thalamus | Missing | V |
| P19 | HIE | MRI abnormal (unspecified) | 1-5-7 | V |
| P20 | HIE, Prematurity (23 weeks) | IVH | 4-7-7 | V |
| P21 | HIE | Thalamus, White matter, Putamen | 0-1-3 | III |
| P22 | HIE | Not specified | Missing | V |
| P23 | HIE | Basal ganglia, Thalamus | 0-3-5 | IV |
| P24 | HIE, Prematurity (34 weeks) | White matter | 1-8-10 | II |
| P25 | HIE, Prematurity (32 weeks) | MRI abnormal (unspecified) | Missing | III |
| P26 | Prematurity (34 weeks), fetofetal transfusion syndrome (donor), neonatal sepsis | Basal ganglia | 6-7-7 | V |
| APGAR: Appearance Pulse Grimace Activity Respiratory effort, DCP: Dyskinetic Cerebral Palsy, HIE: Hypoxic Ischemic Encephalopathy, IVH: Intraventricular Hemorrhage, CFCS: Communication Function Classification System | | | | |

| **Supplemental table 1b**. FDA-2 total, sections and category scores for patients with DCP and ID/IN before DBS. | | | | | |
| --- | --- | --- | --- | --- | --- |
|  |  | Total (n=26) | DCP (n=14) | ID/IN (n=12) | p-value |
| FDA-2 | | | | | |
| Total score | Mean (SD) | 46.3 (33.6) | 29.3 (21.6) | 66.1 (35) | 0.005 |
|  | Median | 38 | 21.5 | 58.5 |  |
|  | IQR | 18.5 to 63.5 | 14 to 39 | 41 to 103 |  |
|  | Min - max | 9.5 to 104 | 9.5 to 87.5 | 15 to 104 |  |
| FDA-2 sections | | | | | |
| Reflexes | Mean (SD) | 7.7 (3.5) | 6.8 (2.7) | 8.7 (4.1) | 0.185 |
|  | Median | 8 | 7.3 | 11 |  |
|  | IQR | 4.5 to 11 | 4 to 8.5 | 4.8 to 12 |  |
|  | Min - max | 2 to 12 | 2 to 11 | 2 to 12 |  |
| Respiration | Mean (SD) | 4.3 (2.9) | 2.3 (2.1) | 6.5 (1.7) | <0.001 |
|  | Median | 4 | 3 | 7 |  |
|  | IQR | 3 to 7 | 0 to 4 | 5.5 to 8 |  |
|  | Min - max | 0 to 8 | 0 to 7 | 3.5 to 8 |  |
| Lips | Mean (SD) | 9.2 (6.8) | 5.9 (5.1) | 13 (6.7) | 0.007 |
|  | Median | 6.5 | 4.5 | 12 |  |
|  | IQR | 3 to 12.5 | 2.5 to 11 | 6.5 to 20 |  |
|  | Min - max | 0 to 20 | 0 to 17.5 | 3 to 20 |  |
| Palate | Mean (SD) | 7 (3.6) | 5.8 (2.7) | 8.4 (4.1) | 0.074 |
|  | Median | 7 | 5.8 | 9.5 |  |
|  | IQR | 4 to 10 | 4 to 7 | 5.3 to 12 |  |
|  | Min - max | 0 to 12 | 1.5 to 11.5 | 0 to 12 |  |
| Laryngeal | Mean (SD) | 5.5 (6.3) | 2 (3.9) | 9.5 (6.3) | 0.002 |
|  | Median | 2.5 | 0 | 9.3 |  |
|  | IQR | 0 to 10.5 | 0 to 2 | 3.8 to 16 |  |
|  | Min - max | 0 to 16 | 0 to 12 | 0 to 16 |  |
| Tongue | Mean (SD) | 9 (8) | 5.1 (4.6) | 13.5 (8.9) | 0.009 |
|  | Median | 6 | 4 | 10.3 |  |
|  | IQR | 3 to 12.5 | 2 to 6.5 | 5.8 to 23.5 |  |
|  | Min - max | 0 to 24 | 0 to 17.5 | 3 to 24 |  |
| Intelligibility | Mean (SD) | 3.8 (4.9) | 1.4 (3.2) | 6.6 (5.2) | 0.007 |
|  | Median | 0.5 | 0 | 6.8 |  |
|  | IQR | 0 to 7.5 | 0 to 0 | 1.8 to 12 |  |
|  | Min - max | 0 to 12 | 0 to 11 | 0 to 12 |  |
| FDA-2 categories | | | | | |
| Oral structure | Mean (SD) | 7.7 (3.7) | 5.8 (3.8) | 10 (1.7) | 0.001 |
| (at rest) | Median | 8.5 | 6 | 9.5 |  |
|  | IQR | 5 to 11 | 2 to 9 | 8.5 to 12 |  |
|  | Min - max | 0 to 12 | 0 to 11 | 8 to 12 |  |
| Nonverbal | Mean (SD) | 27.5 (18.5) | 19.1 (10.9) | 37.3 (21) | 0.016 |
| oromotor | Median | 22 | 16.8 | 32 |  |
| function | IQR | 12 to 35 | 12 to 26 | 22.5 to 59.5 |  |
|  | Min - max | 6 to 60 | 7.5 to 47 | 6 to 60 |  |
| Motor speech | Mean (SD) | 11 (12.8) | 4.4 (8.7) | 18.8 (12.7) | 0.004 |
| function | Median | 6.8 | 0 | 18.3 |  |
|  | IQR | 0 to 19.5 | 0 to 4 | 9.8 to 31.8 |  |
|  | Min - max | 0 to 32 | 0 to 29.5 | 0 to 32 |  |

^a^ p-values are from Welch’s t-test
Abbreviations: SD standard deviation, IQR inter quartile range, 1^st^ to 3^rd^ quartile

**Supplemental table 1c**. FDA-2 total, sections and category scores for patients with DCP and ID/IN 12 months after DBS.

|  |  | Total (n=26) | DCP (n=14) | ID/IN (n=12) | p-value^a^ |  |
| --- | --- | --- | --- | --- | --- | --- |
| FDA-2 | | | | | |  |
| Total score | Mean (SD) | 46.3 (34.2) | 28.4 (22) | 67.1 (34.7) | 0.004 |  |
|  | Median | 37 | 20 | 65 |  |  |
|  | IQR | 17 to 74 | 14 to 39 | 37 to 103.5 |  |  |
|  | Min - max | 8.5 to 104 | 8.5 to 87.5 | 17 to 104 |  |  |
| FDA-2 sections |  |  |  |  |  |  |
| Reflexes | Mean (SD) | 7.8 (3.3) | 6.7 (2.7) | 9.2 (3.6) | 0.069 |  |
|  | Median | 8 | 7 | 11 |  |  |
|  | IQR | 6 to 10.5 | 4 to 8.5 | 7 to 12 |  |  |
|  | Min - max | 2 to 12 | 2 to 10.5 | 2 to 12 |  |  |
| Respiration | Mean (SD) | 4.1 (2.8) | 2.3 (2.1) | 6.3 (1.8) | <0.001 |  |
|  | Median | 4 | 3 | 7 |  |  |
|  | IQR | 3 to 6.5 | 0 to 4 | 4.3 to 8 |  |  |
|  | Min - max | 0 to 8 | 0 to 6.5 | 3.5 to 8 |  |  |
| Lips | Mean (SD) | 9.7 (7) | 5.9 (5) | 14 (6.5) | 0.002 |  |
|  | Median | 7 | 4 | 17 |  |  |
|  | IQR | 4 to 17 | 2 to 11 | 6.5 to 20 |  |  |
|  | Min - max | 0 to 20 | 0 to 17 | 5 to 20 |  |  |
| Palate | Mean (SD) | 6.8 (3.8) | 5.3 (2.9) | 8.7 (3.9) | 0.023 |  |
|  | Median | 7 | 4 | 10 |  |  |
|  | IQR | 4 to 10 | 3.5 to 7 | 7 to 12 |  |  |
|  | Min - max | 0 to 12 | 0 to 11.5 | 0 to 12 |  |  |
| Laryngeal | Mean (SD) | 5.1 (6.2) | 2 (3.9) | 8.6 (6.6) | 0,007 |  |
|  | Median | 2 | 0 | 8 |  |  |
|  | IQR | 0 to 9,5 | 0 to 2 | 2,3 to 16 |  |  |
|  | Min - max | 0 to 16 | 0 to 12 | 0 to 16 |  |  |
| Tongue | Mean (SD) | 9.1 (8.3) | 4.9 (5) | 14 (8.9) | 0.006 |  |
|  | Median | 6 | 3 | 13 |  |  |
|  | IQR | 3 to 16 | 2 to 6.5 | 5.3 to 23.5 |  |  |
|  | Min - max | 0 to 24 | 0 to 19 | 3.5 to 24 |  |  |
| Intelligibility | Mean (SD) | 3.7 (4.8) | 1.4 (3.2) | 6.3 (5.1) | 0.010 |  |
|  | Median | 1 | 0 | 5 |  |  |
|  | IQR | 0 to 6.5 | 0 to 0 | 1.5 to 12 |  |  |
|  | Min - max | 0 to 12 | 0 to 11 | 0 to 12 |  |  |
| FDA-2 categories | | | | | |  |
| Oral structure | Mean (SD) | 7.9 (3.8) | 5.7 (3.9) | 10.4 (1.6) | <0.001 |  |
| (at rest) | Median | 9 | 6 | 11 |  |  |
|  | IQR | 4.5 to 11 | 2 to 9 | 9 to 12 |  |  |
|  | Min - max | 0 to 12 | 0 to 11 | 8 to 12 |  |  |
| Nonverbal | Mean (SD) | 27.7 (18.8) | 18.4 (11.5) | 38.5 (20.2) | 0.007 |  |
| oromotor | Median | 22 | 14 | 37 |  |  |
| function | IQR | 12 to 43.5 | 12 to 26 | 22.3 to 59.5 |  |  |
|  | Min - max | 5 to 60 | 5 to 47.5 | 8 to 60 |  |  |
| Motor speech | Mean (SD) | 10.8 (12.8) | 4.4 (8.6) | 18.3 (13.1) | 0.006 |  |
| function | Median | 5 | 0 | 18 |  |  |
|  | IQR | 0 to 20.5 | 0 to 4 | 6.3 to 32 |  |  |
|  | Min - max | 0 to 32 | 0 to 29 | 0 to 32 |  |  |
| ^a^ p-values are from Welch’s test  Abbreviations: SD standard deviation, IQR inter quartile range, 1st to 3rd quartile | | | | | | |

| **Supplementary Table 1d.** Gpi-BDS Stimulation Paramenters of the included Patients. | | | | | | |
| --- | --- | --- | --- | --- | --- | --- |
| ID | Gpi Left Contacts | Gpi Right Contacts | Pulse Width (µs) | Frequency (Hz) | Gpi Left Amplitude (mA) | Gpi Right Amplitude (mA) |
| P01 | 1, 2 | 9, 10 | 70 | 154 | 1,2 | 2,5 |
| P02 | 2, 3 | 10, 11 | 110 | 74 | 4 | 4 |
| P03 | 1, 2 | 9, 10 | 120 | 130 | 2,5 | 2,1 |
| P04 | 1, 2 | 9, 10 | 120 | 130 | 3,4 | 3,4 |
| P05 | 2 | 10 | 90 | 130 | 2,7 | 2,9 |
| P06 | 1, 2 | 9, 10 | 120 | 130 | 2,4 | 2,4 |
| P07 | 1, 2, 3, 4 | 9, 10, 11, 12 | 130 | 120 | 3 | 3 |
| P08 | 1, 2 | 9, 10 | 130 | 120 | 2,3 | 2,3 |
| P09 | 1, 2, 3, 4 | 9, 10, 11, 12 | 60 | 130 | 1,5 | 1,5 |
| P10 | 1, 2, 3, 4 | 9, 10, 11, 12 | 120 | 130 | 1,7 | 1,5 |
| P11 | 2, 3, 4 | 10, 11, 12 | 120 | 130 | 2,6 | 2,6 |
| P12 | 2, 3, 4 | 10, 11, 12 | 60 | 130 | 2,6 | 1,5 |
| P13 | 1, 2 | 9, 10 | 120 | 130 | 3,3 | 3,3 |
| P14 | 2 | 10 | 90 | 130 | 2,6 | 2,6 |
| P15 | 1, 2 | 9, 10 | 60 | 130 | 1,6 | 1,6 |
| P16 | 2, 3 | 10, 11 | 60 | 130 | 2,5 | 2,5 |
| P17 | 1, 2 | 9, 10 | 210 | 130 | 1,8 | 1,8 |
| P18 | 1, 2, 3, 4 | 9, 10, 11, 12 | 90 | 130 | 2,7 | 2,7 |
| P19 | 2, 3 | 9, 10 | 90 | 174 | 5 | 5 |
| P20 | 3, 4 | 12 | 90 | 66 | 4,5 | 3,2 |
| P21 | 2 | 10 | 90 | 130 | 3,8 | 3,8 |
| P22 | 1, 2 | 9, 10 | 120 | 130 | 2,9 | 3.0 |
| P23 | 1, 2, 3, 4 |  | 60 | 130 | 3,9 |  |
| P24 | 1 | 9 | 90 | 114 | 4 | 4 |
| P25 | 1, 2 | 9, 10 | 90 | 114 | 1,1/3,4 | 1,1/3,4 |
| P26 | 1, 2 | 9, 10 | 120 | 143 | 3 | 3 |
